# Structures, Interactions, and Antimicrobial Activity of the Shortest Thanatin Peptide from *Anasa tristis*

**DOI:** 10.3390/ijms26199571

**Published:** 2025-09-30

**Authors:** Swaleeha Jaan Abdullah, Jia Sheng Guan, Yuguang Mu, Surajit Bhattacharjya

**Affiliations:** School of Biological Sciences, Nanyang Technological University, Singapore 637551, Singapore; swaleeha003@e.ntu.edu.sg (S.J.A.);

**Keywords:** host defense antimicrobial peptide, thanatin, NMR, LPS, LptA, LptA_m_

## Abstract

Antimicrobial peptides (AMPs), also referred to as host defense peptides, are promising molecules in the development of the next generation of antibiotics against drug-resistant bacterial pathogens. Thanatin comprises a family of naturally occurring cationic AMPs derived from several species of insects. The first thanatin, 21 residues long, was identified from the spined soldier bug, and more thanatin peptides have been discovered in recent studies. The 16-residue thanatin from *Anasa tristis*, or Ana-thanatin, represents the shortest sequence in the family. However, the antimicrobial activity and mechanistic process underpinning bacterial cell killing have yet to be reported for Ana-thanatin peptide. In this work, we examined the antibacterial activity, structures, and target interactions of Ana-thanatin. Our results demonstrated that Ana-thanatin exerts potent antibiotic activity against strains of Gram-negative and Gram-positive bacteria. Biophysical studies demonstrated that Ana-thanatin interacts with LPS outer membrane and can permeabilize the OM barrier in the process. Atomic-resolution structures of the peptide in free solution and in complex with lipopolysaccharide (LPS) micelle were solved by NMR, determining canonical β-sheet structures. Notably, in complex with LPS, the β-sheet structure of the peptide was better defined in terms of the packing of amino acid residues. Further, MD simulations demonstrated rapid binding of the Ana-thanatin peptide with the LPS molecules within the lipid bilayers. These studies have revealed structural features which could be responsible for LPS-OM disruption of the Gram-negative bacteria. In addition, NMR heteronuclear single quantum coherence (HSQC) studies have demonstrated that Ana-thanatin can strongly interact with the LPS transport periplasmic protein LptA_m_, potentially inhibiting OM biogenesis. Taken together, we surmise that the Ana-thanatin peptide could serve as a template for the further development of novel antibiotics.

## 1. Introduction

Since the discovery of penicillin, antibiotics have served as widely employed therapeutics to defend mankind from fatal microbial infections. However, the therapeutic values of old antibiotics are significantly challenged by the emergence and rapid spread of drug-resistant and multi-drug-resistant (MDR) bacterial pathogens [1,2,3]. The initial O’Neill report, the analysis of the World Health Organization, and global works by the Antimicrobial Resistance Collaborator indicate the extraordinary challenges posed by antibacterial resistance in human healthcare [4,5,6]. A more recent study by the Antimicrobial Resistance Collaborator group indicated that in the year 2021 alone, 4.71 million mortalities were associated with bacterial AMR, whereas 1.14 million deaths could be attributable to bacterial AMR globally [7]. Their analyses have further predicted a global estimate of 1.91 million and 8.22 million deaths attributable to, and associated with, AMR in 2050, respectively [7]. Specifically, methicillin-resistant *Staphylococcus aureus*, or MRSA and carbapenems, and fluoroquinolones-resistant *Escherichia coli*, *Klebsiella pneumoniae*, *Acinetobacter baumannii*, and *Pseudomonas aeruginosa* are the leading cause of bacterial AMR deaths [7]. It is noteworthy that these multidrug-resistant Gram-negative bacteria are of serious concern in bacterial AMR. Specifically, Gram-negative bacteria are intrinsically resistant to several front-line antibiotics, e.g., vancomycin, erythromycin, novobiocin, etc., which are otherwise effective in killing Gram-positive pathogens [8,9,10]. There has been a substantial deficit in active druggable molecules that can potentially be developed into antibiotics to treat MDR infections by Gram-negative pathogens [11,12,13].

Antimicrobial peptides (AMPs) are promising lead-molecules in creating the next generation of novel antibacterials against strains of MDR bacteria, including Gram-negative [14,15,16,17,18]. As a mode of action, most AMPs are known to cause bacterial cell lysis by damaging membranes [19,20,21]. The membranolytic activity of AMPs appears to follow one of the following mechanisms: barrel stave, toroidal pore, or carpet [22,23,24]. The LPS outer membrane of Gram-negative bacteria, which is impermeable to many conventional antibiotics, can be disrupted by cationic AMPs [25,26,27,28]. The permeabilization of the OM is a vital process to help in gaining access to the bacterial plasma membrane and intracellular targets [25,26,27,28]. However, such a membranolytic mechanism of cell killing could lack selectivity, limiting therapeutic modalities [29,30,31]. A novel group of cationic AMPs, termed outer membrane protein targeting antibiotics (OMPTA), could perhaps be advantageous in developing therapeutics. These antibiotic peptides can act in a dual mode: binding to lipopolysaccharide (LPS) to disrupt OM integrity and concomitant inhibition of OM biogenesis by targeting proteins [32,33]. OMPTA are bacteria-selective and can inhibit the growth of MDR Gram-negative bacteria, with limited toxicity to humans [32,33].

Thanatins are a group of insect-derived cationic AMPs that are akin to the class of OMPTA [34,35,36,37,38]. The first member of the thanatins group was isolated and characterized from the haemolymph of *Podisus maculiventris* [39]. The 21-residue-long single disulfide-bonded *Podisus maculiventris* thanatin, or Pod-thanatin, and its analogs are the most studied in the family [37,38,39,40,41,42,43]. Recent works have identified thanatin-like AMPs from several other insects [36,44,45]. While analyzing genomic databases, Huynh et al. found several potential thanatin-like AMPs from wide-ranging insect species [44]. Interestingly, their study identified a putative shorter—i.e., 16-residue (V^1^PIYY^5^CNRKT^10^GKCRR^15^L)—thanatin peptide from the quash bug *Anasa tristis* [44]. The primary structure of *Anasa tristis* thanatin (Ana-thanatin) lacks five amino acid residues (GSKKP) in comparison of Pod-thanatin (G^1^SKKP^5^VPIIY^10^CNRRT^15^GKCQR^20^M). Also, there are substitutions of some of the residues, as marked in red. In this work, we have examined Ana-thanatin in terms of its antibacterial activity, LPS outer membrane interactions, atomic-resolution structures, and LptA_m_ binding, with LptA_m_ being a truncated but functional variant of the LptA periplasmic component of the LPS transport protein complex. Despite its shorter length and amino acids variation, Ana-thanatin demonstrated potent antibacterial activity. Furthermore, our study provides structural and mechanistic insights toward Gram-negative bacteria cell killing.

## 2. Results

### 2.1. Antibacterial Activity of Ana-Thanatin

Bacterial growth inhibition by Ana-thanatin was examined for Gram-negative and Gram-positive bacterial strains (Table 1). Ana-thanatin demonstrated low MICs, ranging from 1 to 2 µM against ATCC strains of Gram-negative *Escherichia coli*, *Klebsiella pneumoniae*, and *Salmonella enterica*. Ana-thanatin also inhibited the growth of Gram-positive *Streptococcus pyogenes*; however, MIC was high (>16 µM) for *Enterococcus faecalis* (Table 1).

### 2.2. Ana-Thanatin-Mediated Outer Membrane Permeabilization and Bacterial Surface Charge Neutralization

**Figure 1:**
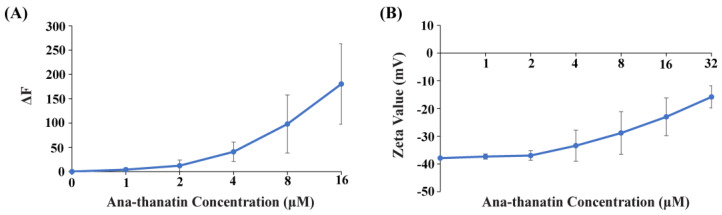
(**A**) Changes in fluorescence emission (ΔF) of NPN as a function of concentrations of Ana-thanatin in *E. coli* cell solutions. Experiments carried out in 10 mM sodium phosphate buffer with bacterial cell density OD_600_ of 0.5. Fluorescence spectra were recorded with an excitation wavelength of 350 nm and an emission of 390–450 nm. (**B**) Peptide dose-dependent changes in ζ potential values of *E. coli* cells.

We examined whether Ana-thanatin can permeabilize the LPS outer membrane and interact with the surface charge of Gram-negative bacteria. Disruption of the LPS outer membrane by Ana-thanatin was probed via NPN fluorescence assays of *E. coli* cells. NPN is impermeable to an undamaged OM; however, it would show enhance fluorescence upon binding with a permeabilized OM. Figure 1A shows an increase in fluorescence (ΔF) at the emission maxima of NPN upon additions of Ana-thanatin in solutions of E. coli cells, suggesting permeabilization of the LPS outer membrane. The binding of Ana-thanatin to the LPS-OM of *E. coli* cells was probed by measuring the ζ potential (Figure 1B). The OM of Gram-negative bacteria is highly negatively charged compared to that of Gram-positive due to the phosphate and carboxylate groups of the LPS. The ζ potential values of *E. coli* cells were found to increase upon addition of Ana-thanatin, suggesting neutralization of the bacterial surface charge due to its binding to the LPS outer membrane (Figure 1B).

### 2.3. NMR Analyses of Ana-Thanatin in Free Solution and in Complex of the LPS Micelle

**Figure 2:**
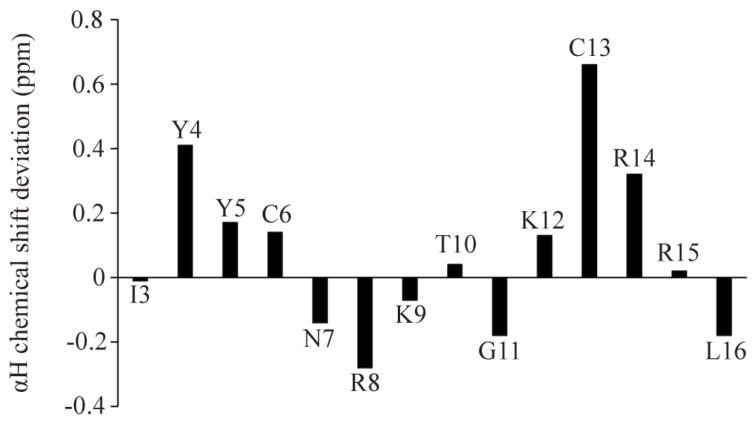
Bar diagram showing the secondary chemical shift of the ^α^H resonances of each amino acid of Ana-thanatin.

**Figure 3:**
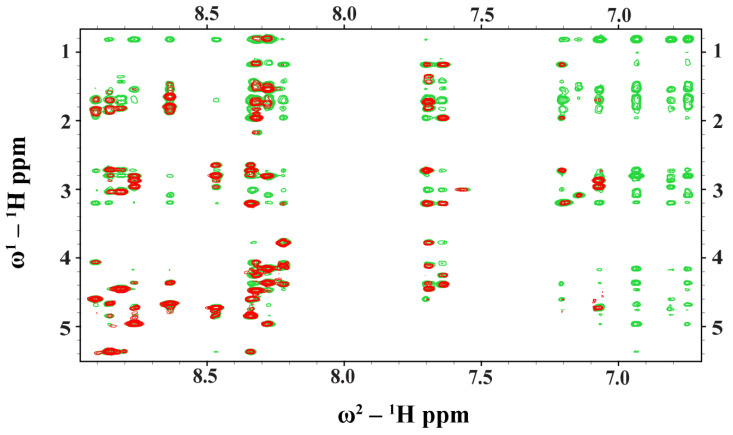
Overlay of partial ^1^H-^1^H two-dimensional NOESY (red contour) and tr-NOESY (green contour) spectra of Ana-thanatin peptide. NOESY/tr-NOESY spectra showing NOEs involving downfield-shifted amides and aromatic proton resonances along the w_2_ dimension, with the upfield-shifted aliphatic proton resonances along the w_1_ dimension. Clearly, more NOEs were detected in the presence of the LPS micelle.

NMR spectroscopy serves as the most common method of determining the atomic resolution structures of AMPs in solution and in solid states [46,47,48,49,50]. Analyses of two-dimensional ^1^H-^1^H TOCSY and ^1^H-^1^H NOESY spectra were utilized to obtain the sequence-specific resonance assignments of individual amino acids of Ana-thanatin in free solution. Figure 2 shows the secondary chemical shift [51] or chemical shift deviation from a random coil of ^α^H resonances of amino acid residues of the Ana-thanatin peptide. As seen, residues Y4, Y5, C6, K12, C13, and R14 delineate positive deviation values, suggesting β-strand conformations for these residues, whereas negative secondary shift values were estimated for residues N7, R8, K9, and G11 (Figure 2). Two-dimensional ^1^H-^1^H NOESY and tr-NOESY spectra of Ana-thanatin in free solution and in complex with LPS micelle showed marked differences in terms of the number of NOEs detected (Figure 3). Ana-thanatin demonstrated a far greater number of NOEs in complex with LPS micelles. Table 2 summarizes the long-range NOE contacts that could be detected for both Ana-thanatin in free solution and in complex with the LPS outer membrane. There were only six long-range NOEs for the peptide in solution, whereas in complex with LPS micelles, as many as 19 long-range NOEs were observed. Observation of more long-range NOEs in complex with LPS micelle indicates that the peptide’s structure could be better folded in comparison to its free state.

### 2.4. Atomic-Resolution Structures of Ana-Thanatin in Free Solution and in Complex with LPS Outer Membrane

**Figure 4:**
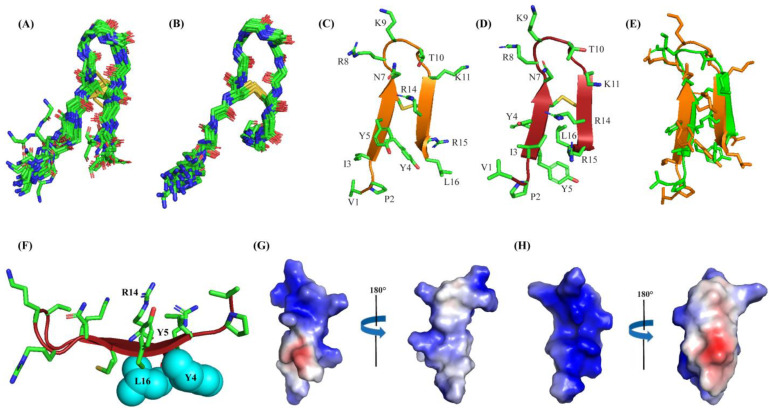
(**A**) Superposition backbone atoms (αC, N, C’) of twenty low-energy structures of Ana-thanatin in free solution. (**B**) Superposition backbone atoms (αC, N, C’) of twenty low-energy structures of Ana-thanatin in complex with LPS micelle. (C, D) Ribbon representation of structure of Ana-thanatin in free solution (**C**) and in complex with the LPS micelle (**D**), showing sidechain orientation and backbone topology. (**E**) Superposition of structures of Ana-thanatin in free solution (green ribbon) and in complex of the LPS micelle (orange ribbon). (**F**) Cation-π interactions between sidechains of residue Y5 and R14 and non-polar packings between sidechains of residues Y6 and L16 in the β-hairpin structure of Ana-thanatin determined in complex with LPS micelle. (**G**,**H**) Electrostatic potential surface (blue: positive charge; red: negative charge; white: neutral) of Ana-thanatin structure in free solution (**G**) and in complex with LPS micelle (**H**). Structural coordinates of Ana-thanatin in free solution and in complex with LPS micelles are provided in Supplementary Materials S1 and S2, respectively.

Structures of Ana-thanatin were determined in both a free state and in LPS micelle-bound complex by CYANA. Figure 4A,B delineates the backbone (Cα, N, C′) superpositions of twenty low-energy structures of Ana-thanatin in free form (Figure 4A) and as a complex of LPS outer membrane (Figure 4B). The parameters of the structural ensembles are listed in Table 3. Structural ensembles of Ana-thanatin are superposed with backbone and all the heavy atoms, with RMSD values of 0.57 Å and 1.06 Å, respectively, whereas the RMSD values of backbone and all the heavy atoms of the free structures were estimated to be 1.22 Å and 2.11 Å, respectively. Regardless, the backbone topology of the β-hairpin structure, canonical to disulfide boned thanatins, can be seen for Ana-thanatin both in free solution (Figure 4C) and in the LPS outer membrane complex (Figure 4D). The superposition of both the free and LPS-bound structures of Ana-thanatin revealed differences in sidechains and backbone orientations (Figure 4E). Notably, the atomic structure of Ana-thanatin in complex with the LPS outer membrane demonstrated well-defined sidechain interactions across the two anti-parallel β-stands of the β-hairpin fold. The aromatic sidechain of residue Y5 is in close proximity with the sidechain of cationic residue R14, potentially indicating cation/π packing interactions (Figure 4F). Furthermore, non-polar packing can be realized between the aromatic sidechain of residue Y4 and the alkyl sidechain of residue L16 (Figure 4F). It may be noted that residues R14 and L16 are substituted by Q and M, respectively, in the Pod-thanatin sequence. In other words, these inter-side chain interactions observed in complex with LPS could be considered novel and specific to the Ana-thanatin peptide. Further, the β-hairpin structure in the LPS outer membrane of Ana-thanatin demonstrated a well-defined and focused larger cationic surface compared to the structure in free solution (Figure 4G,H).

### 2.5. MD Simulation Studies of Ana-Thanatin with LPS Dipalmitoylphosphatidylethanolamine (DPPE) Lipid Bilayer

**Figure 5:**
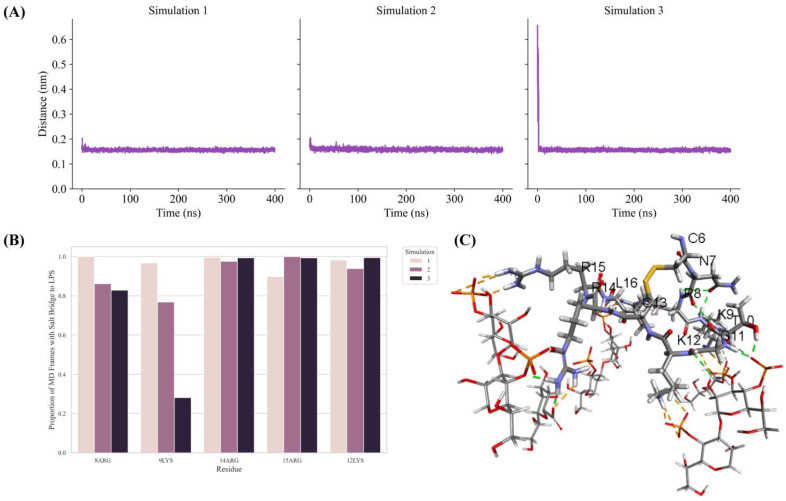
(**A**) Plots show minimum distance between Ana-thanatin and the LPS in the MD simulations. The minimum distance decreased quickly and remained stable throughout the three MD simulations. (**B**) Bar diagram showing potential ionic interactions identified in the three MD simulations between residues of Ana-thanatin and LPS molecules in the lipid bilayer. A large proportion of the MD simulation trajectories were found with ionic interactions occurring between Ana-thanatin and LPS molecules. (**C**) The largest cluster among the MD simulations were extracted for non-bonding interaction analysis. A representative cluster obtained through clustering using the linkage method with cutoff set to 0.1 nm. The orange and green dotted lines represent ionic interactions and hydrogen bonds, respectively. The thick and thin lines represent Ana-thanatin and LPS, respectively. Only the residues found with interactions with LPS molecules are shown. Structural coordinates of Ana-thanatin/LPS bilayer are provided in Supplementary Materials S3.

To gain better insights into peptide–LPS interactions, 400 ns MD simulations were performed using the LPS outer membrane structure of Ana-thanatin and a lipid bilayer consisting of the LPS and DPPE lipids. Note that DPPE has been used in constructing the LPS–lipid bilayer in a previous work involving pod-thanatin [40]. The minimum distance between the Ana-thanatin structure and the LPS in the bilayer was observed to be reduced rapidly and remained stable during the course of the three MD simulations (Figure 5A). Analyses of three MD trajectories revealed that most of the cationic residues of the β-hairpin structure of Ana-thanatin could be involved in potential salt bridge or ionic interactions with the negatively charged phosphate groups of the LPS molecules (Figure 5B). Figure 5C delineates a representative structure of the LPS–Ana-thanatin complex, demonstrating ionic and hydrogen bonding interactions in the MD simulation.

### 2.6. Binding of Ana-Thanatin with LptA_m_ and Alpha-Fold3-Derived Structure of the Complex

**Figure 6:**
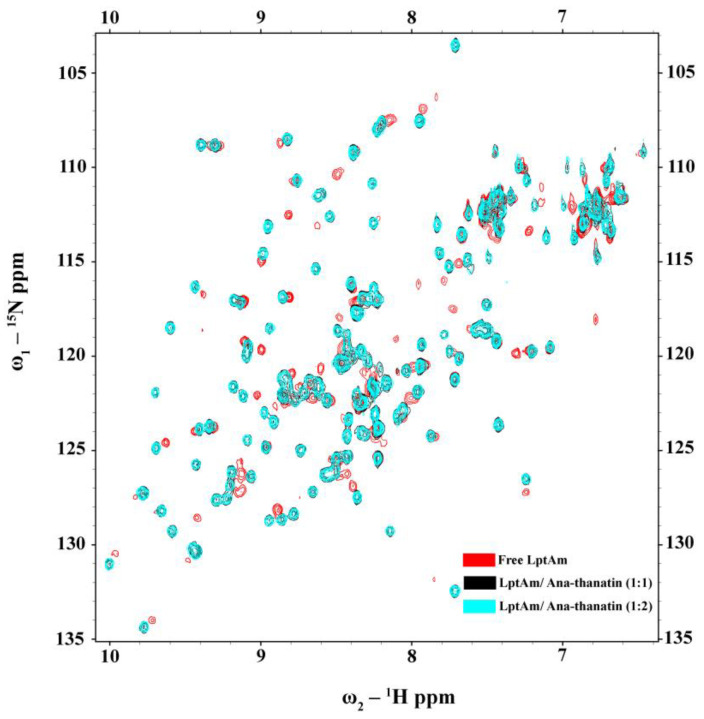
^15^N-^1^H HSQC titration of ^15^N-LptA_m_ in the free form (red contour) and in the presence of Ana-thanatin at molar ratios of 1:1 (black contour) and 1:2 (cyan contour).

**Figure 7:**
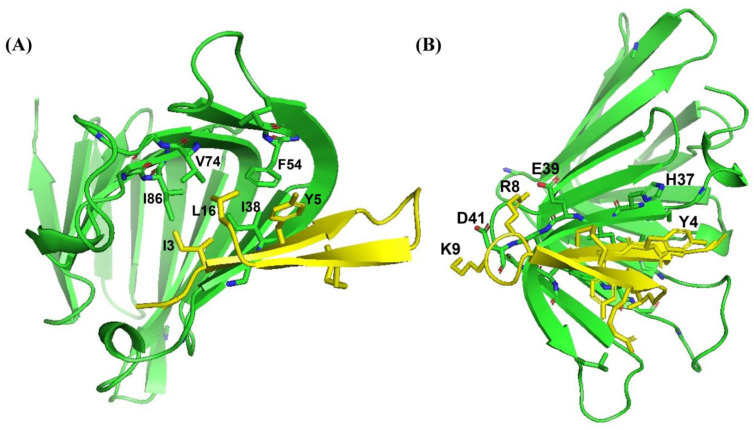
AlphaFold3-derived model structure of the complex of LptA_m_ and Ana-thanatin peptide showing (**A**) sidechain packings and (**B**) polar interactions. The structural coordinates of the Ana-thanatin–LptA_m_ complex bilayer are provided in Supplementary Materials S3.

As a dual mode of action, thanatin peptides target the protein components of the seven-protein complex with the Lpt system (LptA-G) involved in transporting LPS to OM. Thanatins from *P. maculiventris* (Pod-thanatin), *C. ubica*, and *M. histrionica* are known to bind to the periplasmic protein of *E. coli* LptA or LptA_m_, inhibiting the transport of the LPS molecules to the biogenesis of bacterial OM [37,44]. Pod-thanatin also binds to the LptD of the complex [52]. We examined by use of NMR heteronuclear single quantum coherence (HSQC) whether the shorter Ana-thanatin peptide can interact with LptA_m_ protein. Figure 6 shows ^15^N-1H HSQC spectra of LptA_m_ of *Klebsiella pneumoniae* at 1:0 (in red), 1:1 (in black), and 1:2 (in cyan) molar ratios of Ana-thanatin. The ^15^N-^1^H HSQC spectra of free LptA_m_ were well dispersed in ^1^H and ^15^N chemical shifts, typifying a folded protein. Inclusions of Ana-thanatin peptide at a 1:1 ratio caused significant changes in terms of both ^1^H and ^15^N chemical shifts in the ^15^N-^1^H HSQC spectra of LptA_m_, demonstrating binding interactions (Figure 6). Notably, no further changes in HSQC spectra could be observed at a 1:2 protein–peptide molar ratio, suggesting a tight 1:1 complex. AlphaFold3 was used to generate a model of the LptA_m_–Ana-thanatin complex (Figure 7). In the complex, the N-terminal hydrophobic residues I3 and Y5 of the β-hairpin structure of Ana-thanatin demonstrated potential interactions with the binding pocket residues of LptA_m_, akin to other thanatins (Figure 7A) [37,44]. The alkyl sidechain of residue L16 of Ana-thanatin was also positioned at the non-polar pocket of the LptA_m_. In addition, potential ionic interactions can be seen in the complex involving residues R8/E39 and K9/D41 (Figure 7B). The aromatic sidechain of residue Y4 of Ana-thanatin is in close proximity to the sidechain of residue H37 of LptA_m_ (Figure 7B).

## 3. Discussion

Thanatins are a unique group of insect-derived AMPs which have significant potential for novel antibiotic discovery, particularly against MDR Gram-negative bacteria [34,37,38,43]. The 21-residue Pod-thanatin, the founding member of the family, from *Podisus maculiventris*, has been investigated extensively, defining the structure–activity relationship, the atomistic structures in the LPS-OM complex, and targeted bacterial proteins [40,42,44]. Recently, thanatin peptides have been found from various species of insects [44,45]. However, the newly discovered thanatins are yet to be fully investigated. Here, we have examined the shortest (16-residue) naturally occurring Ana-thanatin from Anasa tristis, or squash bug. Most of the thanatins reported so far have been 20 or 21 amino acids long. Our study demonstrated that despite the shorter length, Ana-thanatin possesses potent antibacterial activity against Gram-negative bacteria such as *Escherichia coli*, *Salmonella enterica*, and *Klebsiella pneumoniae*. The peptide also showed growth inhibition of the Gram-positive strain *Streptococcus pyogenes*; however, it showed limited activity with respect to *Enterococcus faecalis*. Ana-thanatin demonstrated the LPS outer membrane permeabilization and surface charge alternation of bacterial cells. The atomic-resolution structures of Ana-thanatin—both as a free peptide and in complex with the LPS outer membrane—are solved by NMR spectroscopy. The backbone folding of the free peptide was determined to be of a β-hairpin structure, akin to that of longer thanatins. The 3D structure of Ana-thanatin in complex with the LPS outer membrane maintains the monomeric β-hairpin topology. However, in contrast to the free solution conformation, striking differences were observed in the dispositions or orientations of the sidechains of many amino acids in the LPS outer membrane structure of Ana-thanatin. Interestingly, a dimeric four-stranded anti-parallel β-sheet structure was determined for the Pod-thanatin (GSKKPVPIIYCNRRTGKCQRM) in complex with LPS micelle [40,53]. Several long-range NOEs involving residues at the N-terminus of the β-harpin were observed in the dimeric structure of Pod-thanatin [40,53]. However, the tr-NOESY spectra of Ana-thanatin were devoid of such long-range NOEs in complex with the LPS outer membrane. Notably, the LPS micelle-bound structures of 16-residue N-terminal truncated analogs of Pod-thanatin delineated a monomeric β-hairpin structure [54,55,56]. These truncated analogs of Pod-thanatin also exhibited antibacterial activity comparable to the parent peptide [54,55,56]. Therefore, interactions of N-terminal residues G^1^SKKP^5^ with LPS could perhaps be essential in stabilizing the higher-order structure of native Pod-thanatin. Regardless, we further probed LPS–Ana-thanatin peptide interactions by using MD simulations studies in the LPS-DPPE lipid bilayer. Residues R8, K9, K11 located at the loop and residues R14 and R15 at the β-strand of the β-hairpin structure appeared to be pivotal to the binding interactions with the negatively charged head groups of the LPS molecules (Figure 5B,C). In the bilayer, cationic residues of Ana-thanatin were able to form ionic or salt bridge interactions with multiple LPS molecules. Apart from these, sidechains of residues Y5, N7, and L16 were found to be in close proximity with the LPS layer. The binding interactions of the peptide with LPS could perhaps be the first step in causing the destabilization of the LPS outer membrane to a permeabilized OM. As a mode of Gram-negative bacterial cell killing, thanatins, including Pod-thanatin and analogs, are known to disrupt OM biogenesis by inhibiting LPS transport across periplasmic space. Based on the structures and functional analyses, it has been proposed that the binding of Pod-thanatin either to full-length LptA or LptA_m_ (a truncated but functional nonaggregating analog of LptA) can cause the disruption of protein–protein interactions involving heteromeric LptC-LptA or homomeric LpA/LptA in periplasm within the Lpt transport complex [41,57,58]. We have assessed the ^15^N-^1^H HSQC-NMR binding interactions of synthetic Ana-thanatin peptide with ^15^N-labeled LptA_m_, which demonstrated a tight 1:1 complex of LptA_m_–Ana-thanatin. However, determination of the 3D structure of the complex is yet to be completed. Here, we presented a model structure of the LptA_m_–Ana-thanatin complex deduced by AlphaFold3. In the complex of LptA_m_, the mode of binding the β-hairpin structure of Ana-thanatin is highly analogous to that of pod-thanatin and other thanatin peptides. The sidechains of residues I3 and Y5 are inserted into the hydrophobic pocket of the β-sheet structure of LptA_m_. In addition, the cationic sidechains of residues R8 and K9 from the loop of the β-hairpin structure of the peptide are in potential ionic and/or hydrogen bonding interactions with residues E39 and D41 of LptA_m_, respectively. The LptA_m_–Ana-thanatin complex is likely to inhibit the LPS translocation process, resulting in bacterial cell death.

## 4. Materials and Methods

### 4.1. Peptide and Chemicals

The Ana-thanatin peptide was chemically synthesized by GenScript^TM^, Piscataway, NJ, USA. The molecular weight of the peptide was confirmed by mass spectroscopy. NPN, LPS-O111:B4, and Muller–Hinton broth were purchased from Sigma Aldrich^TM^, Saint Louis, MI, USA. Ni-NTA affinity resin was obtained from QiagenTM Singapore. Bacterial strains were procured from American Type Culture Collection (ATCC)TM, Manassa, VA, USA. All other fine chemicals were of analytical grade.

### 4.2. Determination of Antibacterial Activity of Ana-Thanatin

Bacterial growth inhibition assays were carried out to determine the Minimal Inhibitory Concentration (MIC) of the Ana-thanatin peptide. Three Gram-negative strains, namely, Escherichia coli ATCC 25922, Salmonella enterica ATCC 14028, and Klebsiella pneumoniae ATCC 13883; and two Gram-positive strains, namely, Streptococcus pyogenes ATCC 19615 and Enterococcus faecalis ATCC 29212, were used for the assays. Overnight culture solutions of bacteria were grown until a mid-log phase (OD_600_~0.4), and cell solutions were then diluted in a 2X Mueller Hinton (MH) broth to a final OD_600_ of 0.002. In a 96-well plate, 100 μL of the diluted bacterial solutions and 100 μL of Ana-thanatin peptide concentrations ranging from 0.5 μM to 16 μM, diluted from 100 μM of stock Ana-thanatin peptide in MilliQ water (measured by spectrophotometer), were incubated at 37 °C for 18 h. For the positive control, 100 μL of MilliQ water and 100 μL of diluted bacterial culture were incubated, whereas for the negative control, 200 μL of MH broth was added. MIC assays were repeated three times on separate days.

### 4.3. LPS Outer Membrane (LPS-OM) Permeabilization Assay

Steady-state fluorescence emission of 1-N-phenylnapthylamine (NPN) was used to probe the LPS-OM permeabilizing activity of the Ana-thanatin peptide of *E. coli* cells. Fluorescence experiments were conducted using a Cary Eclipse spectrophotometer (Varian, Palo Alto, CA, USA) in a 0.1 cm quartz cuvette with an excitation wavelength of 350 nm, whereas emissions were recorded from 390 to 450 nm. NPN (10 mM) fluorescence emission spectra were obtained in solutions of 10 mM sodium phosphate buffer containing E. coli cells, with density adjusted to an OD_600_ of 0.5 from a mid-log phase culture. NPN fluorescence experiments were performed in the absence of and in the presence of various doses (1 to 16 µM) of the Ana-thanatin peptide.

### 4.4. Zeta (ζ) Potential Measurements

Bacterial surface charge neutralization by the Ana-thanatin peptide was assessed by measuring the ζ potential with a Zeta Sizer Nano ZS (Malvern Instruments, Worcestershire, UK) instrument equipped with a 633 nm He laser. For these experiments, mid-log phase E. coli cultures were harvested by centrifugation at 5000 rpm for 10 min, and solutions were prepared in 10 mM sodium phosphate buffer, pH 7. The ζ potentials of bacterial cell suspensions were estimated from 1 to 32 μM concentrations of Ana-thanatin in zeta cells with gold electrodes. In each measurement, 100 runs were performed with three replicates.

### 4.5. Recombinant Expression and Purification of LptA_m_

A synthetic gene of LptA_m_, coding for amino acids 28–159 of Klebsiella pneumoniae, subcloned into a pET24a(+) plasmid vector, was commercially obtained (GenScriptTM, Piscataway, NJ, USA) for recombinant protein production in *E. coli* BL21 cells. The expressed protein contains a six-histidine tag at the C-terminus for Ni-NTA affinity purification. Expression and purification of LptA_m_ was carried out following previous protocol [53,54]. Briefly, for production of the ^15^N-labeled LptA_m_ protein, plasmid-transformed BL21 cells were grown in M9 minimal medium supplemented with 15N-ammonium chloride. Bacterial cells were induced by 1 mM isopropyl β-d-1-thiogalactopyranoside (IPTG) at OD600~0.6 at 18 °C for 18 h for protein expression. Cells were harvested by centrifugation at 6000 RPM, 4 °C, for 15 min. Cell pallets were resuspended in a buffer (100 mM HEPES, 500 mM NaCl, 10 mM imidazole, pH 8) and sonicated at 25 Amp for 1 h on ice. The cell supernatant was separated after centrifugation at 18,000 RPM, 4 °C, for 30 min, and was passed through Ni-NTA (Qiagen) beads thrice before being washed with increasing concentrations of imidazole. His-tagged LptA_m_ was eluted from the Ni-NTA beads by treating with high imidazole buffer (20 mM HEPES, 150 mM NaCl, 200 mM imidazole, pH 8). Protein samples were further purified by FPLC size-exclusion chromatography in a 50 mM sodium phosphate buffer containing 150 mM NaCl, pH, 6.5.

### 4.6. NMR Studies of Ana-Thanatin Peptide

All NMR data were collected using a Bruker DRX 600 spectrometer (Bruker, Bellerica, MA, USA) maintained with a cryo-probe and pulse field gradients. Raw NMR data were processed using TopSpin and analyzed by SPARKY (T.D. Goddard and D.G. Kneller, University of California, San Francisco, CA, USA). Two-dimensional ^1^H-^1^H TOCSY and NOESY spectra of Ana-thanatin were acquired in 10 mM sodium phosphate buffer, pH 5.8, at 278 K, with mixing times of 80 ms and 200 ms, respectively. Two-dimensional transferred NOESY (tr-NOESY) experiments were performed in the presence of 20 μM LPS at a mixing time 150 ms. A total of 1 mM concentration of DSS (2,2-dimethyl-2-silapentane 5-sulfonate sodium salt) was used as a reference for ^1^H chemical shifts. ^1^H-^15^N HSQC spectra of LptA_m_ were collected at 298 K in free solution and in complex with Ana-thanatin at molar ratios of 1:1 and 1:2.

### 4.7. Structure Calculations of Ana-Thanatin

Atomic-resolution structures of Ana-thanatin in free solution and in complex with LPS outer membrane were determined by CYANA [59]. Distance constraints were qualitatively estimated based on the intensity of NOE cross-peaks, classified as strong, medium, and weak NOEs, with upper bound distance limits of 2.5, 3.5, and 5.0 Å, respectively. The disulfide bond between two Cys residues was maintained during structure calculations. Backbone dihedral (ϕ and φ) constraints were obtained from PREDITOR [60]. Out of 100 structures, 20 structures of lower CYANA target function values were selected for the ensembles and used for further analysis. Stereochemical fitness of the structures were assessed using PROCHECK107 [61].

### 4.8. MD Simulations of Ana-Thanatin in LPS-DPPE Lipid Bilayer

Three independent MD simulations of 400 ns each, were performed using GROMACS 2024.1. Simulations (minimization, equilibration, and production) were prepared using CHARMM-GUI. The lipid bilayer membrane was set up using the membrane builder module of CHARMM-GUI. The top leaflet of the bilayer consisted of 43 LPS molecules, while the bottom leaflet is made up of 138 DPPE lipid molecules. The charge of the system was neutralized by NaCl to a concentration of 150 mM. MD simulations were run at a temperature of 303.15 K. Ana-thanatin peptides were randomly inserted close to the LPS molecules. After the placement of the peptides, the number of Na+ ions were corrected to ensure a neutral system. CHARMM36m forcefield was used for the simulation. To prevent the translocation of the peptide to the lower leaflet, a Lennard-Jones repulsion with a σ of 1 was used between the carbons of the peptide and the carbons of the DPPE molecule near the lipid head. The minimum distance, non-bonded interaction, and structure analysis were performed using Discovery Studio v25.1.0.24284, PyMOL, and MDAnalysis (doi:10.25080/majora-629e541a-00e, PyMOL Molecular Graphics System, Version 3.1.0 Schrödinger, LLC, New York, New York, USA). The plots were generated using the Seaborn package 0.13.2 (https://doi.org/10.21105/joss.03021).

## 5. Conclusions

Thanatins are extremely valuable AMPs for the potential treatment of infections caused by MDR Gram-negative bacteria. This work presents the antibacterial activity, bacterial target interactions, and mechanistic insights for the shortest (i.e., 16-amino acid long) naturally occurring thanatin peptide from *Anasa tristis*, Ana-thanatin. Notably, previous studies have demonstrated that a 16-residue truncated variant of pod-thanatin demonstrates both antibacterial activity and a β-hairpin structure. Taken together, the 16-residue N-terminal segment of thanatins could represent the core antimicrobial region that could be utilized for the further generation of peptidomimetic drug candidates.

## Figures and Tables

**Table 1:** Antimicrobial activity of Ana-thanatin peptide. Minimum inhibitory concentration (MIC, in µM) of the peptide against Gram-negative bacteria, namely, *Escherichia coli* ATCC 25922 (EC), *Salmonella enterica* ATCC 14028 (SE), and *Klebsiella pneumoniae* ATCC 13883 (KP); and Gram-positive bacteria, namely, *Streptococcus pyogenes* ATCC 19615 (SP) and *Enterococcus faecalis* ATCC 29212 (EF).

	MIC (µM)				
	Gram-Negative			Gram-Positive	
	EC	KP	SE	SP	EF
**Ana-thanatin**	2	1–2	1	1	>16

**Table 2:** Long-range NOEs detected for Ana-thanatin in free solution and in the complex of the LPS micelle.

Free Solution	Complex of the LPS Micelle
Y4^δ^H_s_-L16H	I3^δ^Hs-R14^Ɛ^Hs
C6^α^H-R14H	I3^δ^Hs-R15^Ɛ^Hs
C6^β^Hs-R14H	Y4^δ^Hs-R15H
C13^α^H-N7H	Y4^δ^Hs-L16H
R15^β^H_s_-Y4^δ^H_s_	C6^α^H-R14H
R15^α^H-Y5H	C6^β^Hs-R14H
	C13^α^H-N7H
	R14^δ^Hs-Y5^δ^Hs
	R14^δ^Hs-Y5^Ɛ^Hs
	R14^γ^Hs-Y5^δ^Hs
	R14^γ^Hs-Y5^Ɛ^Hs
	R15^α^H-Y4^δ^Hs
	R15^α^H-Y4^Ɛ^Hs
	R15^β^Hs-Y4^δ^Hs
	R15^β^Hs-Y4^Ɛ^H3
	R15^δ^Hs-Y5^δ^Hs
	L16^γ^Hs-Y4^δ^Hs
	L16^γ^Hs-Y5^δ^Hs
	L16^δ^Hs-C6H

**Table 3:** Summary of structural statistics of Ana-thanatin in free solution and in LPS complex.

	Free	LPS
**Distance Constraints**		
Intra residue [|i-j| = 0]	65	82
Sequential [|i-j|= 1]	29	46
Medium range [1 < |i-j| < 4]	5	10
Long range [|i-j| ≥ 4]	6	19
Total NOE	105	158
**Dihedral—angle constraints**	24	24
**Deviation from mean structure**		
Backbone atoms (Å)	1.22	0.57
All heavy atoms (Å)	2.11	1.06
**Ramachandran plot for the mean** **structure**		
% of residues in most favored region and additional allowed region	100	100
% of residues in generously allowed region	0	0
% of residues in disallowed region	0	0

## Data Availability

The original contributions presented in this study are included in the article and Supplementary Materials. Further inquiries can be directed to the corresponding author(s).

## Supplementary Materials

The following supporting information can be downloaded at: https://www.mdpi.com/article/10.3390/ijms26199571/s1.
